# Monolithic Magneto-Optical Nanocomposites of Barium Hexaferrite Platelets in PMMA

**DOI:** 10.1038/srep11395

**Published:** 2015-06-12

**Authors:** Gregor Ferk, Peter Krajnc, Anton Hamler, Alenka Mertelj, Federico Cebollada, Miha Drofenik, Darja Lisjak

**Affiliations:** 1 University of Maribor, Faculty of Chemistry and Chemical Engineering, 2000 Maribor, Slovenia; 2 University of Maribor, Faculty of Electrical Engineering and Computer Science, 2000 Maribor, Slovenia; 3 Jožef Stefan Institute, Department for Complex Matter, 1000 Ljubljana, Slovenia; 4 Universidad Politécnica de Madrid, POEMMA-CEMDATIC, ETSI de Telecomunicación, 28040 Madrid, Spain; 5 Jožef Stefan Institute, Department for Materials Synthesis, 1000 Ljubljana, Slovenia

## Abstract

The incorporation of magnetic barium hexaferrite nanoparticles in a transparent polymer matrix of poly(methyl methacrylate) (PMMA) is reported for the first time. The barium hexaferrite nanoplatelets doped with Sc^3+^, i.e., BaSc_0.5_Fe_11.5_O_12_ (BaHF), having diameters in the range 20 to 130 nm and thicknesses of approximately 5 nm, are synthesized hydrothermally and stabilized in 1-butanol with dodecylbenzenesulfonic acid. This method enables the preparation of monolithic nanocomposites by admixing the BaHF suspension into a liquid monomer, followed by *in-situ*, bulk free-radical polymerization. The PMMA retains its transparency for loadings of BaHF nanoparticles up to 0.27 wt.%, meaning that magnetically and optically anisotropic, monolithic nanocomposites can be synthesized when the polymerization is carried out in a magnetic field. The excellent dispersion of the magnetic nanoparticles, coupled with a reasonable control over the magnetic properties achieved in this investigation, is encouraging for the magneto-optical applications of these materials.

The development of polymer-based (nano)composites that incorporate inorganic (nano)particles into organic polymers makes it possible to adjust their mechanical, thermal, electrical, optical and magnetic properties, depending on the intrinsic properties of the inorganic material^1^,^2^,^3^. In this work we used a polymer matrix of poly(methyl methacrylate) (PMMA) as an amorphous, optically pure, thermoplastic material; this material is usually applied as a substitute for inorganic glass when elastic behaviour rather than brittle fracture is preferred^4^. The chosen nanoparticles in this case were barium hexaferrite platelets. Barium hexaferrite, with a basic composition of BaFe_12_O_19_, is one of the most commonly used materials in hard-magnetic, magnetic recording and high-frequency applications^5^,^6^. However, novel applications for barium hexaferrite have been found in multifunctional (nano)composites, i.e., magnetic photocatalysis, magneto-optics, magneto-electrics, magnetic gels, and ferromagnetic fluids^7^,^8^,^9^,^10^,^11^,^12^. Magnetic materials, in general, can be exploited in magneto-optical (MO) applications, such as recording, sensors and switches, imaging and in a variety of other optical components, like isolators, circulators and modulators^13^,^14^,^15^,^16^. Although it has been demonstrated that optical and magnetic properties could be coupled in magnetic nanoparticles, they cannot be used directly as such^17^. For example, advanced patterned MO media are produced by lithography^18^,^19^. Alternatively, composites produced from magnetically aligned monodomain nanoparticles in a transparent matrix, i.e., liquid crystal^12^, can be prepared using a simple chemical approach. Similarly, PMMA can be used as a transparent matrix, while the alignment of the magnetic nanoparticles can be induced prior to the polymerization and glass formation by applying an external magnetic field. It is of crucial importance to retain the transparency of the polymer after the incorporation of the nanoparticles; this transparency can be lost due to Rayleigh scattering from large nanoparticles or clusters. The intensity of this Rayleigh scattering is proportional to d^6^, where d is the diameter of the nanoparticle/cluster^20^. Therefore, only fully dispersed nanoparticles with d ≤ 30 nm can lead to a polymer composite that is transparent, i.e., without any significant loss of transparency.

Nanoparticles in general, and magnetic nanoparticles in particular, tend to aggregate and form clusters as a consequence of the attractive van der Waals and magnetic dipole forces that act between individual nanoparticles in a monomer liquid prior to the polymerization. In order to minimize these attractive interactions the nanoparticles must be separated by a large enough distance. However, the preparation of dispersions in a given monomer, in which the selected magnetic nanoparticles are individually and homogeneously dispersed, remains as a major challenge. There are only scarce data about transparent nanocomposites of iron oxide magnetic particles in a PMMA matrix^2^,^21^,^22^. The nanoparticles must interact with a selected monomer and therefore their surface chemistry needs to be properly designed. For example, the monomer methyl methacrylate (MMA) exhibits a good miscibility with iron oxide nanoparticles coated with oleic acid, but agglomeration can also occur during the polymerization step. *In-situ*, bulk free-radical polymerization is a method that can overcome the difficulties related to aggregation and makes it possible to prepare pure and optically clear polymer composites^23^,^24^. In this contribution we report, for the first time, about a synthesis procedure that allows for the integration of magnetic monodomain barium hexaferrite nanoparticles with the nominal composition of BaSc_0.5_Fe_11.5_O_12_ (BaHF), and with an anisotropic, plate-like shape, into a polymer composite exhibiting a MO response.

## Results and Discussion

**Figure 1:**
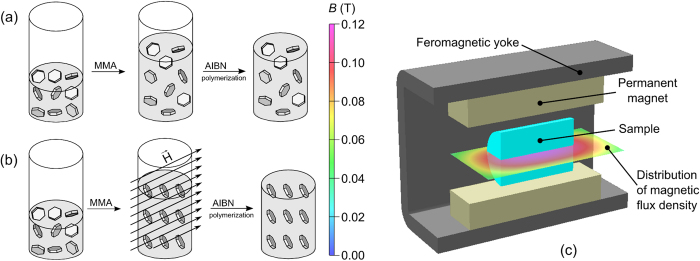
Schematic representation of the *in-situ* polymerization method for the synthesis of BaHF/PMMA nanocomposites. Isotropic (**a)** and anisotropic (**b**) nanocomposites, where a magnetic yoke (**c**) was used to maintain the magnetic field during the polymerization.

**Figure 2:**
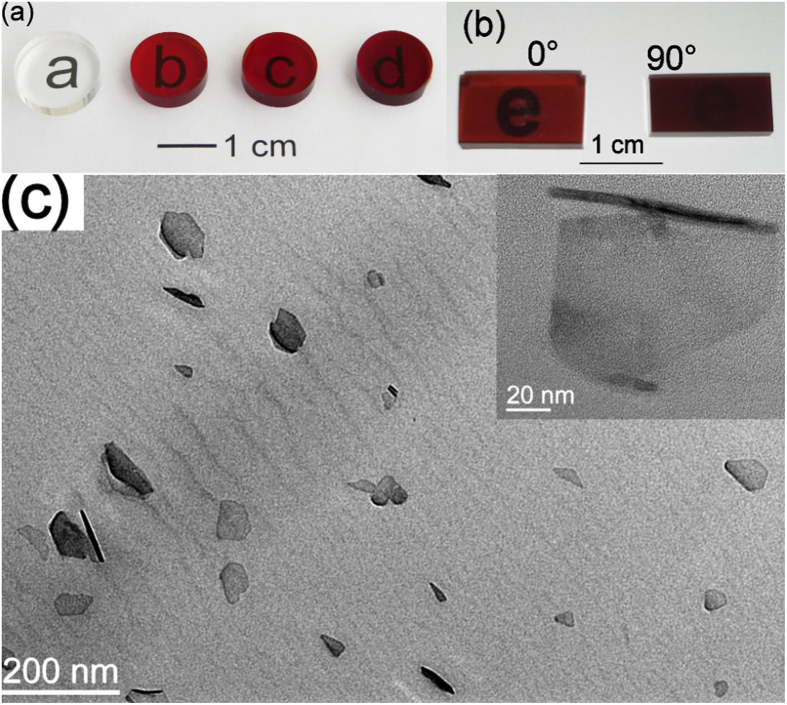
BaHF/PMMA monolithic nanocomposites: photographs of nanocomposites. Isotropic (**a**) and anisotropic (**b**), with thickness of 3 mm and using different loadings (denoted as in Table 1) of BaHF nanoparticles with a typical TEM image of the sample D (**c**). The angles in (**b**) denote the orientation with respect to the basal plane of the aligned nanoplatelets in the PMMA matrix. The inset in (**c**) shows a magnified view of the grain couple with an orthogonal orientation.

The nanocomposites (Table 1) were prepared by the *in-situ*, bulk free-radical polymerization of a dispersion of BaHF nanoparticles in monomer MMA (Fig. 1a). The magnetically oriented sample E was prepared by carrying out the polymerization in a magnetic field (Fig. 1b,c). Fig. 2a,b show BaHF/PMMA monolithic nanocomposites with different nanoparticle loadings in the polymer matrix. The slight red-brownish colour of the samples intensifies for the larger nanoparticle loadings. Nevertheless, all the composites were transparent. The transparency of the monolithic nanocomposites samples with a thickness of 3 mm was retained even with a loading of 0.27 wt.%, when looking in plane with aligned BaHF platelets of the anisotropic sample E (angle 0°in Fig. 2b).

Three steps were necessary for the synthesis of the transparent BaHF/PMMA nanocomposites:

1. The synthesis of sufficiently small magnetic nanoparticles to prevent, or at least minimize, the Rayleigh scattering, which was achieved by applying a hydrothermal synthesis. The BaHF nanoparticles were in the form of thin hexagonal platelets (see Supplementary, Figure S1), typical for barium hexaferrite. Their diameters were in the range of 20 to 130 nm while their thickness did not vary significantly with the diameter and was around 5 nm. As suggested from our previous study^25^, the Fe^3+^ was partly substituted with Sc^3+^ (BaSc_0.5_Fe_11.5_O_12_) in order to narrow the particle-size distribution, while, at the same, keeping their size above the superparamagnetic limit. The selected-area electron diffraction (SAED; see Supplementary, inset in Figure S1a) indicates the barium hexaferrite structure with intense hk0 diffraction rings since the platelets were preferentially aligned in the plane of the supporting grid. These nanoparticles exhibit a room-temperature magnetization of 32 emu g^−1^ (measured at 1 kOe) and a coercivity of 1250 Oe.
2. The preparation of a stable suspension comprising these particles, which was achieved with the electrosteric stabilization of the BaHF nanoparticles in 1-butanol^26^. Monodomain BaHF nanoparticles, dispersed in 1-butanol, were already sensitive to a weak magnetic field. The BaHF platelets oriented collectively in the direction of the magnetic field via rotation in a suspension, causing a visual effect: the translucency of the suspension changed with the direction of the magnetic field (see Supplementary, movie S1). This would not be possible, however, if the platelets were agglomerated.
3. A stable magnetic suspension was prepared in order to provide good miscibility with the liquid monomer (MMA) and subjected to controlled polymerization without any significant aggregation of the nanoparticles to form a transparent, brownish-coloured nanocomposite (Fig. 2a,b). A transmission electron microscope (TEM) image of the nanocomposite sample D with 0.18 wt.% of nanoparticles is shown in Fig. 2c. Except for a few binary aggregates (see an example in the inset of Fig. 2c) only well-dispersed BaHF nanoplatelets were observed after a careful inspection of the sample.

**Figure 3:**
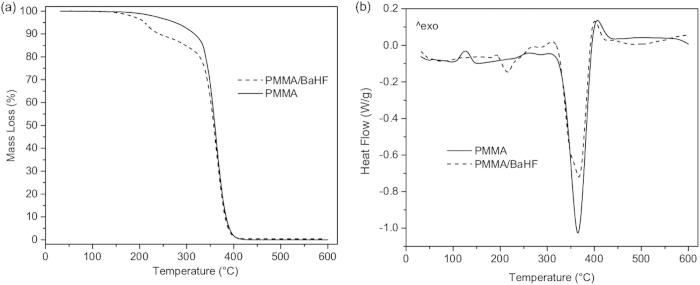
TGA (**a**) and DSC (**b**) analyses data of PMMA and BaHF/PMMA nanocomposite E in a N_2_ atmosphere.

A thermogravimetric analysis (TGA) elucidated the ultimate polymerization phase and the stability of the samples. During the polymerization process the majority of 1-butanol from the original suspension evaporates; however, a minor fraction remains in the polymer matrix. This can be concluded from the first step in the mass loss (Fig. 3a; 160–225 °C), which is only observed in the BaHF/PMMA nanocomposite sample. The differential scanning calorimetry (DSC) measurement supports this by means of a significant endothermic peak at 213 °C (Fig. 3b). The final large step in the mass loss (364 °C) accounts for the total mass loss of PMMA. The DSC measurement (Fig. 3b) was performed to monitor any modifications of the intrinsic properties of the polymeric matrix due to the interactions of the BaHF nanoparticles with the polymer matrix. The DSC curve of the pure PMMA has no distinct glass-transition temperature. The exothermic peak at 130 °C can be attributed to the cold crystallization of pure PMMA. The small endothermic peaks at 284 °C in the pure polymer and the BaHF/PMMA nanocomposite may relate to the dissociations of polymeric chains^27^. The major endothermic peak at 364 °C is attributed, in both samples, to the final decomposition of the PMMA.

**Figure 4:**
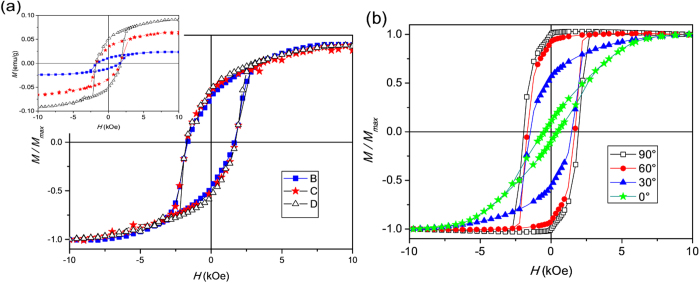
Hysteresis loops, measured at room temperature, of the: isotropic BaHF/PMMA nanocomposites (**a**) and anisotropic nanocomposite E (**b**), measured at different angles between the applied field and the basal plane of the nanoparticle. The inset in (**a**) shows the variation of the mass magnetization with a different nanoparticle loading.

The hysteresis loops of the BaHF/PMMA nanocomposites are shown in Fig. 4a. Note that the diamagnetic contribution of the PMMA to the magnetization was negligible. The values of the magnetization measured at *H* = 10 kOe can be considered to be good approximations to the saturation magnetization (*Ms*), since the slopes of the curves are negligible at *H* > 8 kOe. As expected, the *Ms* and the remanent magnetization (*Mr*) values increase with the increasing loading of the BaHF nanoparticles (see the inset in Fig. 4a). However, the *Mr/Ms* ratio and the coercivity (*Hc*) values are the same for all the samples, indicating the absence of a magnetic interaction between the nanoparticles in the composites.

The nanoplatelets in sample E (Fig. 2b) were oriented during the polymerization (Fig. 1b,c) with their basal planes perpendicular to the applied field and, in such a way, a magnetically anisotropic composite was formed. This was confirmed by the magnetic measurements (Fig. 4b). Here, the magnetic field was applied at different angles with respect to the orientation of the platelets. When the magnetic field is applied perpendicular to the basal planes (90°) of the BaHF platelets, the composite exhibits an essentially square hysteresis loop with a *Mr/Ms* ratio close to one (*Mr/Ms* ∼ 0.9) and a *Hc* value similar to that of the non-oriented composites (*Hc* ∼ 1900 Oe). In contrast, when the applied field is parallel to the basal planes (0°) of the BaHF platelets the magnetization increases almost linearly with the field (essentially constant susceptibility) until the point of saturation, with very low *Mr* and *Hc* values. This indicates that the easy (hard) magnetocrystalline axis of the nanoparticles is perpendicular (parallel) to the basal plane of the platelets. The high *Mr/Ms* ratio for 90° is a consequence of the high degree of orientation of the nanoplatelets in the PMMA matrix. The slight deviation from a perfect orientation is likely due to the presence of a limited number of platelet clusters (Fig. 2c), which is also reflected in a small hysteresis of the 0°-curve. Namely, the almost linear behaviour of the hysteresis curve for 0°, with the low *Mr* and *Hc*, is characteristic of quasi-reversible rotations of the magnetization from the easy axis to the field direction.

**Figure 5:**
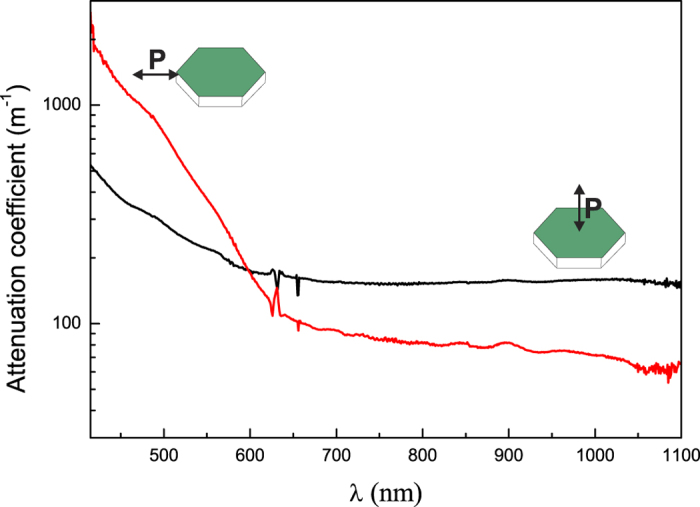
Attenuation coefficient as a function of the wavelength for ordinary (red) and extraordinary (black) polarized light in sample E with a schematic representation of the orientation of the BaHF nanoplatelets during the measurement.

The nanocomposite E is not only magnetically anisotropic; it is also optically anisotropic as can be seen directly in Fig. 2b. The sample is also slightly birefringent (*Δn* ~ 3·10^−4^) and shows a pronounced dichroism, i.e., the attenuation coefficient strongly depends on the polarization of the light (Fig. 5). The measured attenuation coefficients (at the wavelength of 620 nm) are 130 and 170 m^−1^ for the ordinary and extraordinary polarization, respectively. These values are in agreement with the volume concentration of the nanoplatelets in the composite (*Φ* = 0.06%) and the value of the attenuation coefficient measured on a BaHF thin film *α* ~ 2·10^6^ m^−1^ (at 620 nm)^28^, which gives an estimate for the attenuation of *αΦ* ~ 120 m^−1^.

**Figure 6:**
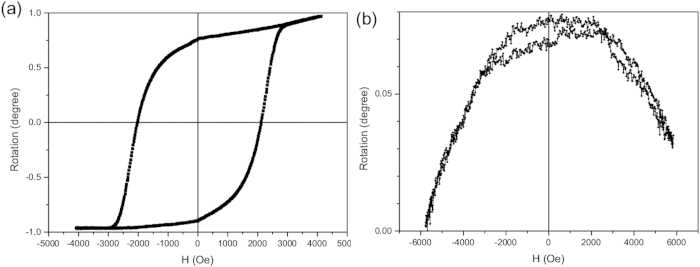
MO properties of the sample E : (**a**) MO hysteresis loop measured with the magnetic field parallel to the easy axis (90°) and (**b**) Faraday rotation measured with the magnetic field parallel to the easy axis (90°).

The MO response of the oriented sample E was checked using a polar configuration, i.e., with the applied field parallel to the incident beam. Prior to the analysis of the composite, the Faraday rotation of a pure PMMA, 1-mm-thick slab was measured. After traversing the PMMA, the polarization of the incident beam rotates at an angle that increases linearly with the field, yielding a Verdet constant of 2.4 ± 1.0·10^−5^ ° Oe^−1^ mm^−1^. Fig. 6a shows the hysteresis loop measured with the field applied perpendicularly to the BaHF platelets, i.e., along the direction of their easy axes, after removing the linear PMMA-induced signal. The respective loop is square-like, with a *Mr/Ms* ratio over 0.8 and a *Hc* value of 2 kOe, which is in agreement with the vibrating-sample magnetometer (VSM) results (Fig. 4b). The Faraday rotation when the magnetization switches from −*Ms* to +*Ms* is close to 2°, i.e., 0.4 ° mm^−1^, which is comparable to the known magneto-optic materials, like for example polycrystalline yttrium-iron garnet films^29^. The slight asymmetry in the loop is probably due to second-order MO effects, which depend on *M*^*2*^. When measuring the MO response with the field parallel to the plane of the nanoplates (i.e., along the hard magneto-crystalline direction) a loop similar to that of the VSM (0° in Fig. 4b), essentially corresponding to reversible rotations of the magnetization from the easy axis to the field direction, should be expected. However, a very weak MO signal was measured (Fig. 6b), with a maximum rotation of the polarization plane below 0.08^o^ when the field is swept from +6 kOe to −6 kOe. The measured signal is composed of a parabolic (quadratic in H) plus a linear contribution. A slight hysteresis is visible in the figure for fields below 3 kOe, coincident with the irreversibilities in the VSM 0^o^ loop. The linear contribution is probably linked to the magnetization of the platelets. The low value of this contribution is due to the small thickness of the platelets (a few nanometres) when they are oriented with their basal planes parallel to the laser beam. This is consistent with the fact that sample E appears darker (lighter) when observed with the planes of the platelets perpendicular (parallel) to the light beam (see Fig. 2b), similar to the suspension of BaHF platelets (see Supplemental, movie S1). The quadratic contribution is likely to be associated with the magneto-mechanical coupling of the nanoparticles to the PMMA matrix. This is because the torque exerted on the platelet’s crystal lattice by the rotation of the magnetization gives rise to mechanical stresses in the PMMA matrix. These stresses are the same, irrespective of the positive or negative orientation of the magnetization, thus producing a quadratic M dependence of the polarization rotation.

In conclusion: The main result of this study is a chemical synthesis procedure for monolithic BaHF/PMMA nanocomposites with a magneto-optical response. The successful admixing of a fully dispersed 1-butanol suspension of BaHF nanoparticles and a liquid monomer prior to the polymerization was the main reason why it was possible to achieve the transparency and homogeneity of the monolithic nanocomposite. The magnetically anisotropic composites were prepared by a magnetic alignment of the BaHF nanoplatelets during the polymerization. This represents the possibility to tailor their magnetic and optical properties. Moreover, the relatively high transparency, significant and anisotropic magneto-optic response of the oriented nanocomposite, and the magneto-mechanical coupling of the BaHF nanoparticles to the PMMA matrix make these nanocomposites technologically interesting for applications such as optical sensors, isolators, modulators, circulators, information storage, as well as magnetic-field and electric-current sensors.

## Methods

### Synthesis

BaHF nanoparticles with the nominal composition BaSc_0.5_Fe_11.5_O_12_ were synthesized hydrothermally and dispersed in 1-butanol using the electrosteric repulsion of the surfactant dodecylbenzene sulphonic acid (DBSa)^26^. In brief, Ba, Fe and Sc nitrates in the atomic ratio Ba:Fe:Sc = 1:4.5:0.5 were dissolved in water and co-precipitated with NaOH. The addition of Sc^3+^ was necessary for the control of the particle size^25^. The slurry was subsequently heated with the DBSa in an autoclave up to 240 °C and cooled down naturally to room temperature. After washing the particles with water, HNO_3_ and acetone, they were dispersed in 1-butanol using ultrasound. The product was centrifuged and the stable supernatant was additionally concentrated in a rotary evaporator to form a stable (no sedimentation during the time of this study) suspension with approximately 1.5 wt.% of nanoparticles. The nanocomposites were prepared by an *in-situ*, bulk free-radical polymerization of the BaHF nanoparticles dispersed in a monomer MMA using 2,2’-azobis(2-methylpropionitrile) (AIBN) as an initiator (Fig. 1). The AIBN was recrystallized from cold methanol and dried at room temperature under vacuum prior to use. The polymerization inhibitor contained in the monomer MMA was removed by alumina column chromatography. In a typical synthesis of the nanocomposite with different nanoparticle loadings (Table 1) a mixture of the BaHF suspension, MMA, and 1-butanol was sonicated for 15 minutes. After that, 0.2 wt.% of the initiator AIBN was added to the solution. The pre-polymerization of the solution was carried out at 75 °C for 20 min under Ar atmosphere. After this, the solution temperature was quickly lowered to the temperature of the main polymerization (45 °C) and maintained for 48 h. Sample E (Table 1) was prepared from a suspension with approximately 3 wt.% of BaHF nanoparticles. In this case, the polymerization was carried out under a magnetic field of about 0.10–0.12 T using a magnetic yoke (Fig. 1b,c).

### Characterization

The dried nanoparticles were characterized using a transmission electron microscope (TEM, Jeol 2100). A drop of diluted suspension was dried on a carbon-coated copper grid under ambient conditions. The equivalent diameters of the nanoplatelets were calculated from their surfaces with Gatan Digital Micrograph Software, while their thicknesses were determined directly from the TEM observations of the platelets, lying perpendicular to the copper grid. The composite sample was trimmed (Leica EM-TRIM2) and thinned (Ultramicrotom Leica EM-UC6) to a thickness of 50 nm for the TEM analysis. The magnetic properties of the nanoparticles and nanocomposites were measured with a VSM (Lake Shore 7407). TGA and DSC were performed with a TGA/DSC (Mettler Toledo) in a N_2_ atmosphere with a flow rate of 100 ml/min. The anisotropy of the absorption (dichroism) was measured using polarized UV-Vis spectroscopy (HP 8453 UV-Vis spectrometer). Birefringence was determined from the changes in the polarization of the laser beam propagating through a thick sample (Uniphase He-Ne laser). The MO characterization of the oriented sample E (with a thickness of 5 mm) was carried out in transmission mode by means of a Faraday set-up in the polar configuration under a maximum applied field of 7 kOe and using a wavelength of 670 nm.

## Additional Information

**How to cite this article**: Ferk, G. *et al*. Monolithic Magneto-Optical Nanocomposites of Barium Hexaferrite Platelets in PMMA. *Sci. Rep*. **5**, 11395; doi: 10.1038/srep11395 (2015).

## Supplementary Material

Supplementary Information

Supplementary Movie S1

## Figures and Tables

**Table 1:** Variable starting ingredients and final loading of the BaHF nanoparticles in the nanocomposite samples.

Sample	MMA[g]	BaHF suspension[g]	1-butanol[g]	BaHF nanoparticles in nanocomposites [wt.%]
A	10.0	0.00	0.00	0.00
B	8.8	0.62	0.58	0.09
C	8.8	0.78	0.42	0.12
D	8.8	1.20	0.00	0.18
E	9.0	1.00	0.00	0.27
