# Supplementary material for: Monolithic Magneto-Optical Nanocomposites of Barium Hexaferrite Platelets in PMMA
Source: Sci Rep. 2015 Jun 12;5:11395. doi: 10.1038/srep11395 (PMC4464329; doi:10.1038/srep11395)
Supplement: Supplementary Information [file srep11395-s1.pdf]

Supplementary Information

Monolithic Magneto-Optical Nanocomposites of Barium Hexaferrite Platelets in PMMA

Gregor Ferk, Peter Krajnc, Anton Hamler, Alenka Mertelj, Federico Cebollada, Miha Drofenik, Darja Lisjak *

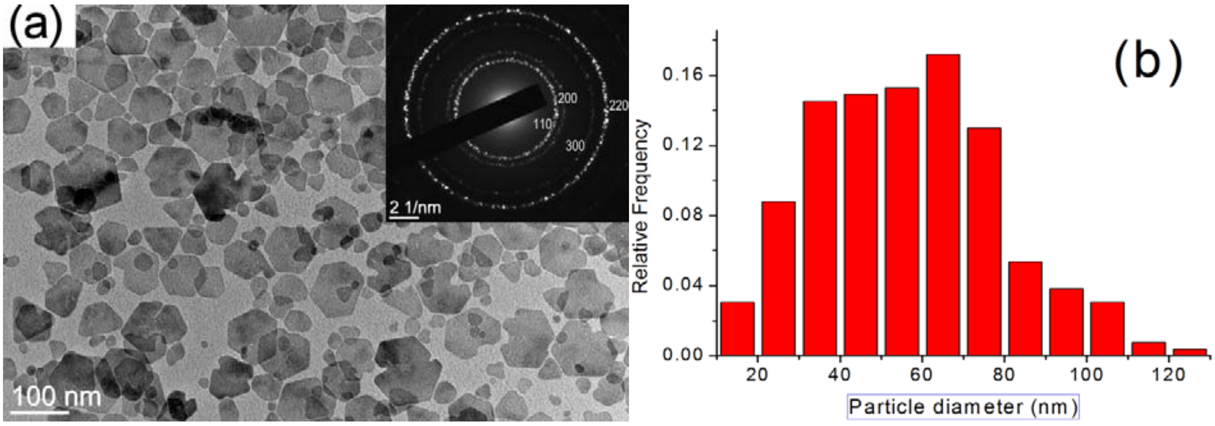

Figure S1. TEM image of dried BaHF nanoparticles from their suspension in 1-butanol with the SAED pattern (a) and the particle size distribution (b). Indices in the SAED correspond to the $P6_3/mmc$ (194) space group.

* Correspondence and requests for materials should be addressed to D. L. (darja.lisjak@ijs.si)
